# ﻿Two new species of the genus *Alloscopus* Börner, 1906 (Collembola, Orchesellidae, Heteromurinae) from southern Thailand

**DOI:** 10.3897/zookeys.1245.148100

**Published:** 2025-07-16

**Authors:** Sopark Jantarit, Nongnapat Manee, Areeruk Nilsai, Natrada Mitpuangchon, Awatsaya Pimsai

**Affiliations:** 1 Excellence Center for Biodiversity of Peninsular Thailand, Faculty of Science, Prince of Songkla University, Hat Yai, Songkhla, 90110, Thailand Prince of Songkla University Songkhla Thailand; 2 Division of Biological Science, Faculty of Science, Prince of Songkla University, Hat Yai, Songkhla, 90110, Thailand Thaksin University Phatthalung Thailand; 3 Faculty of Science and Digital Innovation, Thaksin University, 222, Papayom District, Phatthalung, 93210, Thailand Prince of Songkla University Songkhla Thailand; 4 Princess Maha Chakri Sirindhorn Natural History Museum, Prince of Songkla University, Hat Yai, Songkhla, 90110, Thailand Thaksin University Phatthalung Thailand

**Keywords:** Cave, chaetotaxy, Entomobryoidea, sago palm, taxonomy

## Abstract

Two new species of *Alloscopus* Börner (Orchesellidae: Heteromurinae) are discovered and described from southern Thailand. The first species, *Alloscopussago* Jantarit & Manee, **sp. nov.** was found in a sago palm forest (*Metroxylonsagu* Rottb.), a true sago palm species native to Southeast Asia and typically located in lowland freshwater swamps in Phatthalung Province. The second species, *A.jantapasoae* Jantarit, Nilsai & Manee, **sp. nov.** was sampled from a dark zone within a cave environment in Trang Province. Both species are characterized by the absence of eyes and mucronal spines, the presence of a PAO, two rows of smooth chaetae on the manubrium, and dental spines. However, they differ in several morphological features, including the number of macrochaetae on the ‘A’ series of the head, Th. II and Abd. IV; labial basis chaetotaxy; the presence of smooth chaetae on tibiotarsi; the number of chaetae on both the anterior and posterior ventral tube; and the number of the inter-teeth on the claw. The discovery of these two new species increases the total number of *Alloscopus* species recorded in Thailand to six species with a total of 17 recognized species globally. An updated key to the world species of *Alloscopus* is also provided.

## ﻿Introduction

Currently, the genus *Alloscopus* Börner, 1906 is one of the best-known groups of Heteromurinae. The genus is widespread and is reported to occur in various habitats including canopy, edaphic and subterranean environments. Its distribution is mostly expanded in tropical regions including East Asia to Southeast Asia (India, Thailand, Malaysia, Singapore, Indonesia, Philippines, and southern China) to Oceania (Papua New Guinea, Micronesia, Australia, Hawaii), with a single species recorded in Ecuador and Peru of South America (Mari-Mutt 1977, 1982; Cipola et al. 2016; Jantarit and Sangsiri 2020; Zhang et al. 2020; Alviola et al. 2024). There are 15 known species described under the genus, with a species recently described, *A.arborealis* Alviola, Lucañas & Jantarit, 2024, from Mt. Makiling, Luzon Island, Philippines (Alviola et al. 2024). The genus *Alloscopus* was first erected by Börner in 1906 as a subgenus of *Heteromurus* based on the annulation of antennal segments (Ant.) III and IV and the presence of dental spines. The genus is characterized by having four antennal segments with Ant. I subdivided, Ant. III and IV annulated, head dorsal macrochaetotaxy with S0 present and S2 absent and absence of postsutural and post occipital mac (except Pa5), eyes typically absent, but when present, range from 1+1 to 3+3, post antennal organ (PAO) and dental spines present, manubrium with rows of smooth chaetae (Mari-Mutt 1977, 1982, 1985; Cipola et al. 2016; Jantarit and Sangsiri 2020; Zhang et al. 2020; Alviola et al. 2024).

In Thailand, four species of *Alloscopus* have been reported: *A.tetracanthus* Börner, 1906, and *A.thailandensis* Mari-Mutt, 1985, both found in forest habitats in Northern Thailand (Mari-Mutt 1985; Cipola et al. 2016; Jantarit et al. 2016); and *A.whitteni* Jantarit & Sangsiri, 2020 and *A.namtip* Jantarit & Sangsiri, 2020, both from cave environments in southern Thailand (Jantarit and Sangsiri 2020). Additionally, several undescribed species have been identified in cave habitats (Jantarit et al. 2016).

During a study on the diversity of arthropods in sago palm, led by one of the authors (A. Pimsai) as part of the Plant Genetic Conservation Project under the Royal Initiative of Her Royal Highness Princess Maha Chakri Sirindhorn, an undetermined *Alloscopus* sp. was discovered and collected from a sago palm forest in Phatthalung province, home to the largest sago palm forest in Thailand. Additionally, an undescribed *Alloscopus* sp. was also collected from cave habitats in adjacent area of Trang province. Therefore, in this work, we describe two new species of the genus *Alloscopus* from in southern Thailand from both sago palm forest and cave environment, and present an updated key to the world species of the genus.

## ﻿Materials and methods

Specimens from both sago palm trees and cave environment were collected by using entomological aspirator and modified funnel Berlese extraction. They were sorted and preserved in 95% ethanol and stored in -20 °C in the freezer. Specimens were mounted on slides in Marc-André II medium after clearing in lactic acid. Morphological characters were examined using an Olympus BX51 microscope with phase-contrast. Drawings were made using a drawing tube, and figures were improved with Illustrator CC and were later edited by Adobe Photoshop (Adobe Inc.). Photographs of the habitus were taken by a Canon EOS 6D with Canon EF 100mm f/2.8 Macro lens and optimized by Helicon Remote software. Stacking was performed under Helicon Focus 6. Abbreviations and descriptions follow the conventions of Jantarit and Sangsiri (2020).

**asl.** above sea level;

**Abd.** abdominal segment;

**Ant.** antennal segment;

**Th.** thoracic segment;

**Mac** macrochaeta;

**Mes** mesochaeta;

**Mic** microchaeta;

**sens or S-chaeta** sensillum or sensory seta;

**ms** very short, thick pointed S-chaetae;

**psp** pseudopore;

**tric** trichobotrium;

**tita** tibiotarsus;

**SEM** Scanning Electron Microscope

Dorsal body chaetotaxy follows Szeptycki (1979), and Zhang et al. (2019). Dorsal mac included in the formula are boxed in by dotted line. We use the notation of Mari-Mutt (1977), Ren et al. (2018), and Soto-Adames (2008) for groups of head chaetae; and Fjellberg (1999) for the labial palp. Labial chaetae notation follows Gisin (1967), with the upper-case letter for ciliated and lower-case letter for smooth chaetae. We follow and match the notation system of Jantarit and Sangsiri (2020) for antennal chaetae. The number of dorsal macrochaetae is given from head to Abd. VI. Symbols representing chaetal types used in the figures are as follows: large empty circle = mac; medium empty circle = mes; dark dot = mic; cross (X) = tric; circle with a slash (Ø) = psp.

The holotype and paratypes are deposited at the National Museum of Natural History Philippine National Museum under Philippine National Museum (**NHM-PSU**), Prince of Songkla University (Hat Yai, Songkhla, Thailand).

Animal ethics were approved in accordance with the national guidelines stipulated by the standard of animal research, Research and development office, Prince of Songkla University (Protocol Code: 2564-16-068).

## ﻿Taxonomy

### ﻿Family Orchesellidae Börner, 1906 sensu Godeiro et al. 2023

**Subfamily Heteromurinae Absolon & Kseneman, 1942 sensu Zhang and Deharveng 2015**

**Tribe Heteromurinae Absolon & Ksenemann, 1942**

#### ﻿Genus *Alloscopus* Börner, 1906

##### ﻿ Alloscopus sago

Taxon classificationAnimalia

Jantarit & Manee sp. nov.

0EF6CE3D-6755-5C8F-9334-7918DB4C684B

https://zoobank.org/D25F1316-DD06-4160-B0CD-AD6A6B0369EB

**Figure 1.**
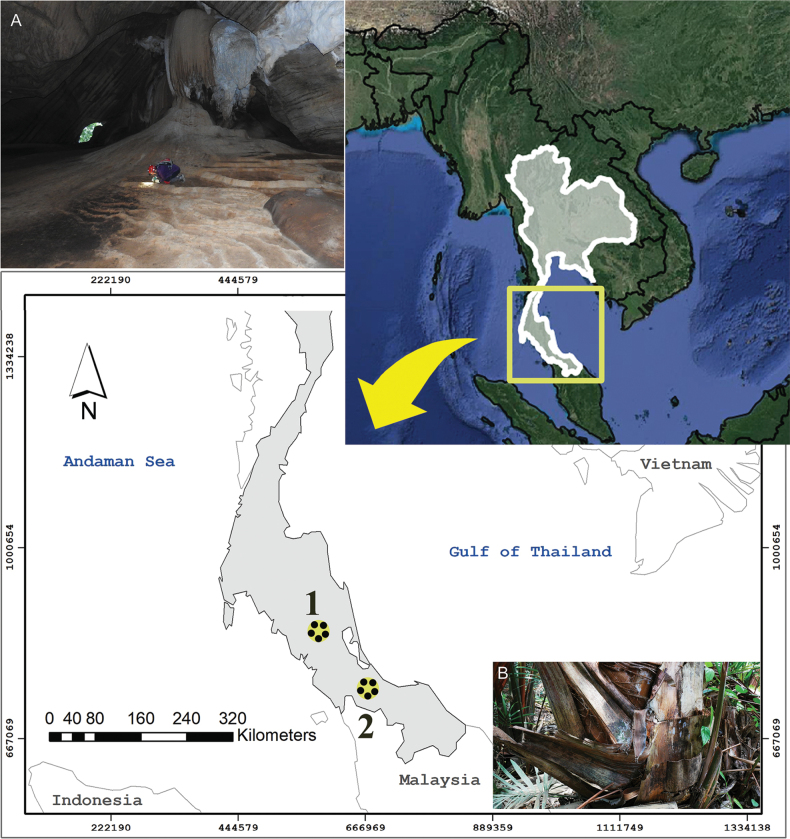
The map of Thailand (top right) shows a rectangle outlining the southern region, with the arrow indicating an enlarged view of this area, illustrating the distribution of the new species of *Alloscopus* described in this study: *Alloscopusjantapasoae* sp. nov. (1); *A.sago* sp. nov. (2); **A.** Photograph of the cave habitat where *A.jantapasoae* sp. nov. was collected, showing a human figure for scale, and **B.** Photograph of the bark of sago palm tree, the habitat where *A.sago* sp. nov. was discovered.

**Figure 2.**
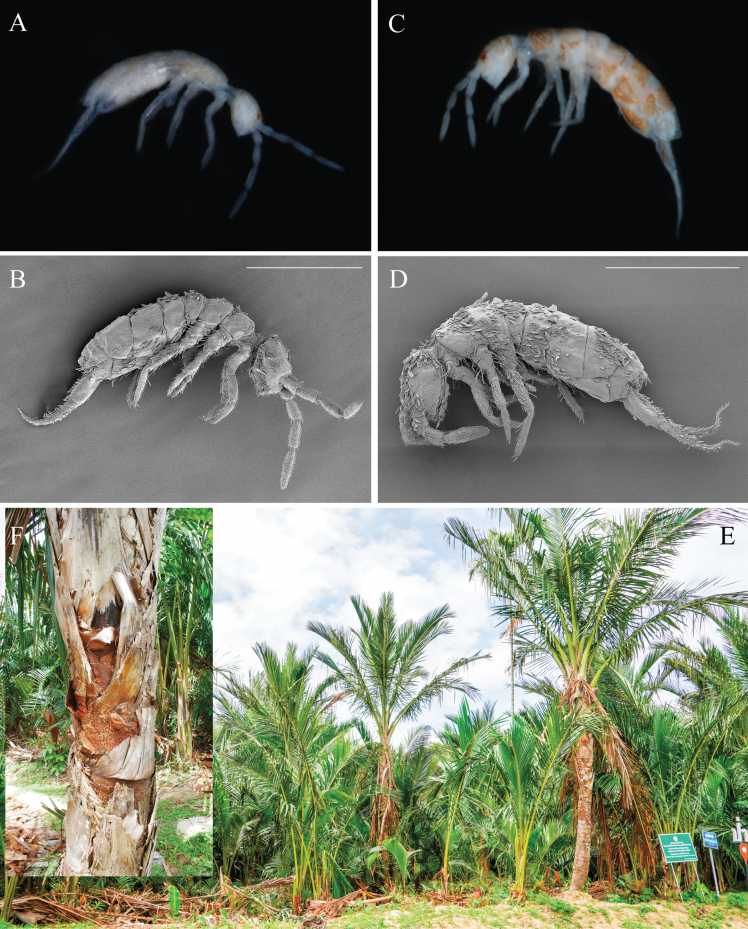
Habitus of *Alloscopus* in this study: **A, B.***A.sago* sp. nov. photographs from the stereomicroscope and SEM and **C, D.***A.jantapasoae* sp. nov. photographs from stereomicroscope and SEM; **E.** The patch of sago palm trees (*Metroxylonsagu* Rottb); **F.** Enlarged detail of sago palm barks. Scale bars: 500 μm (**B, D**).

**Figure 3.**
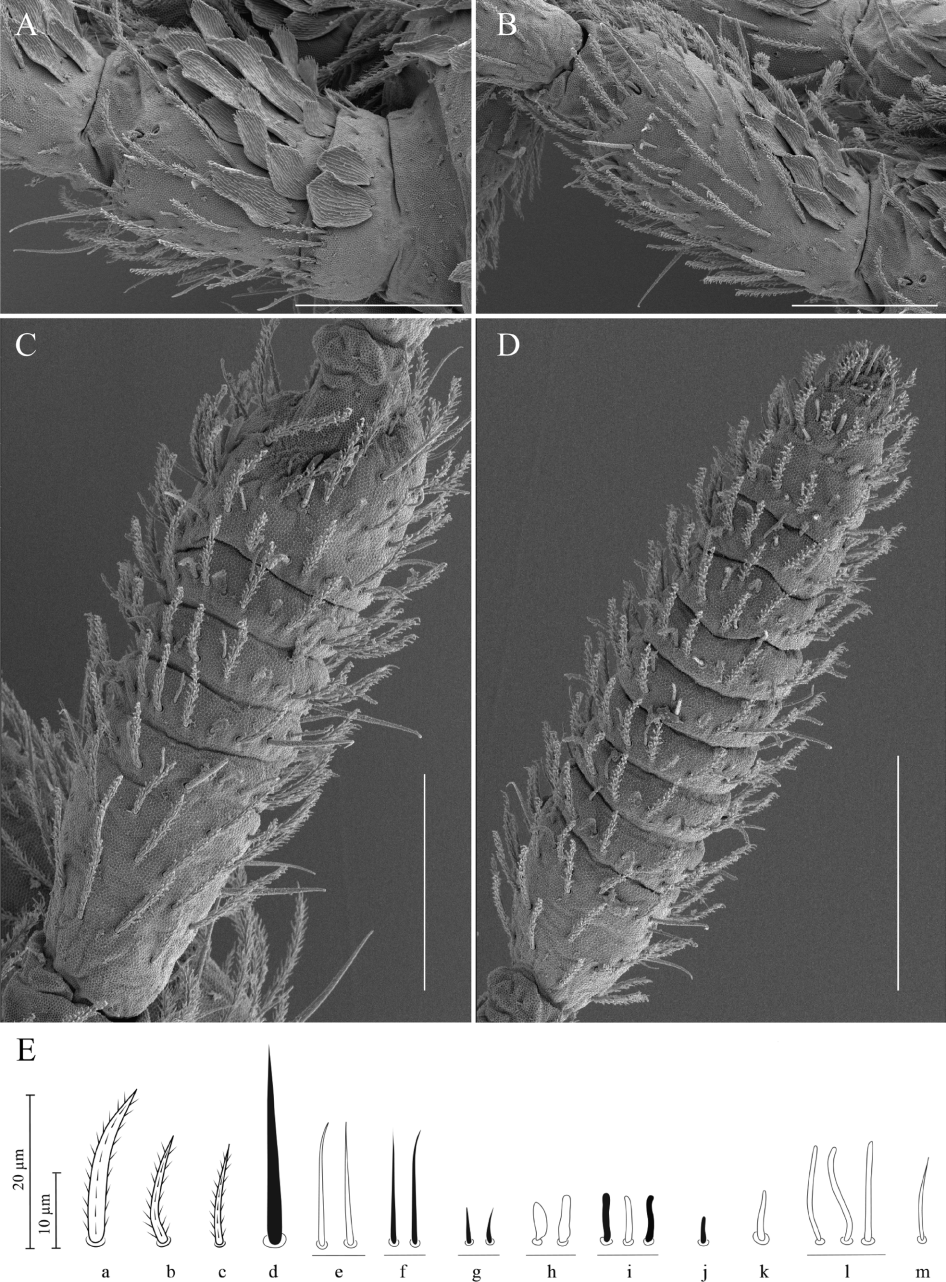
*Alloscopussago* sp. nov. **A.** Dorso-lateral side of Ant. Ia + Ib; **B.** Dorso-lateral side of Ant. II; **C.** Dorso-lateral side of Ant. III; **D.** Dorso-lateral side of Ant. IV; **E.** Antennal chaetae type as per Jantarit and Sangsiri (2020). Scale bars: 40 µm (**A–C**); 50 µm (**D**); 10–20 µm (**E**).

**Figure 4.**
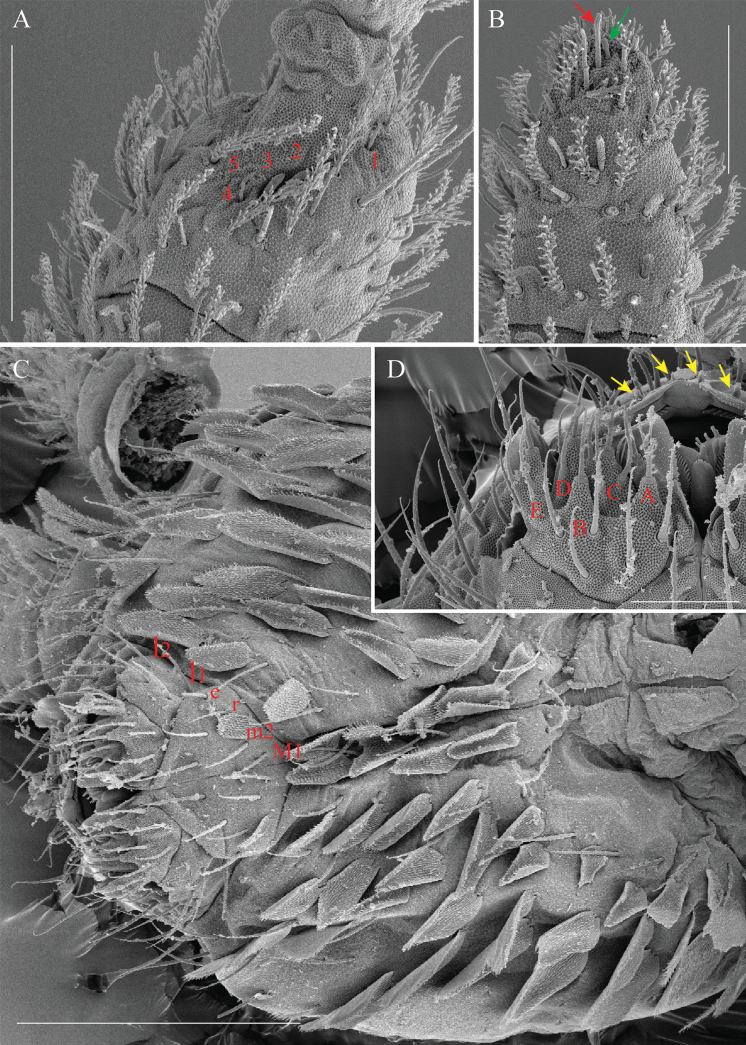
*Alloscopussago* sp. nov. continued, **A.** Antennal Organ III (AOIII) (right side) with its typical 5 chaetae; **B.** Tip of Ant. IV with subapical organite (green arrow) and guard chaeta (red arrow); **C.** Head ventral chaetotaxy anteriorly, focusing on labial basis and PLQ chaetae; **D.** Labial palp of left side, marked with red labels, displays five papillae (**A–E**), while yellow arrows highlight four labral papillae. Scale bars: 40 µm (**A**); 20 µm (**B, D**); 100 µm (**C**).

**Figure 5.**
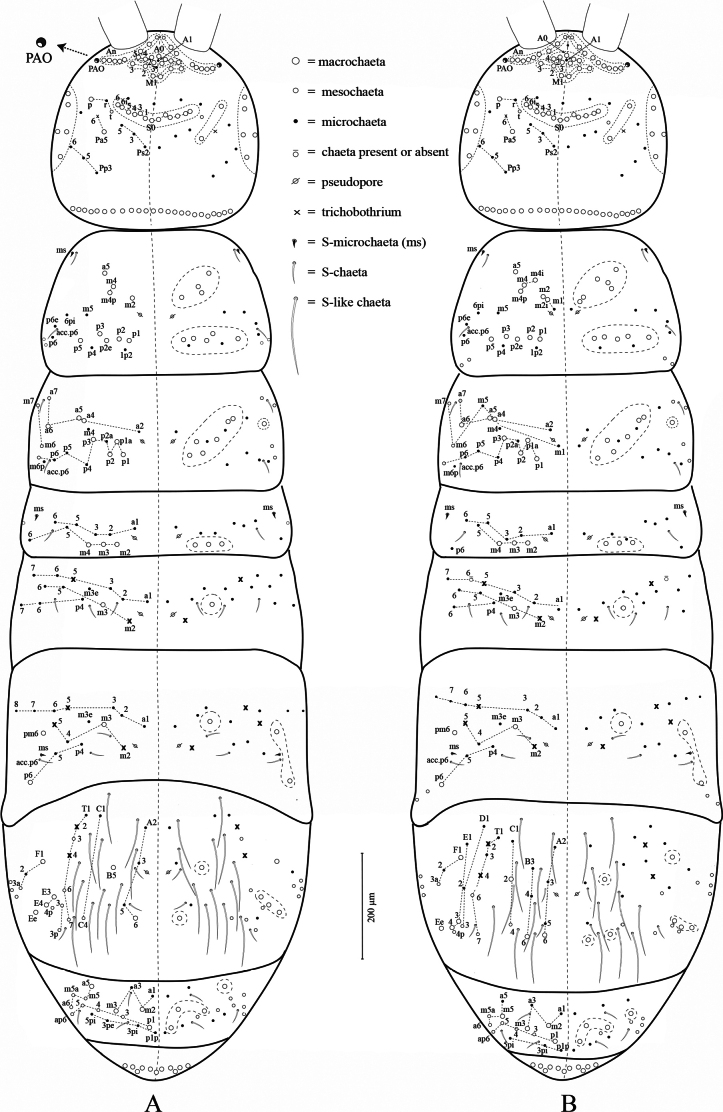
Head and body chaetotaxy of **A.***Alloscopussago* sp. nov. and **B.***A.jantapasoae* sp. nov. Scale bars: 200 µm.

**Figure 6.**
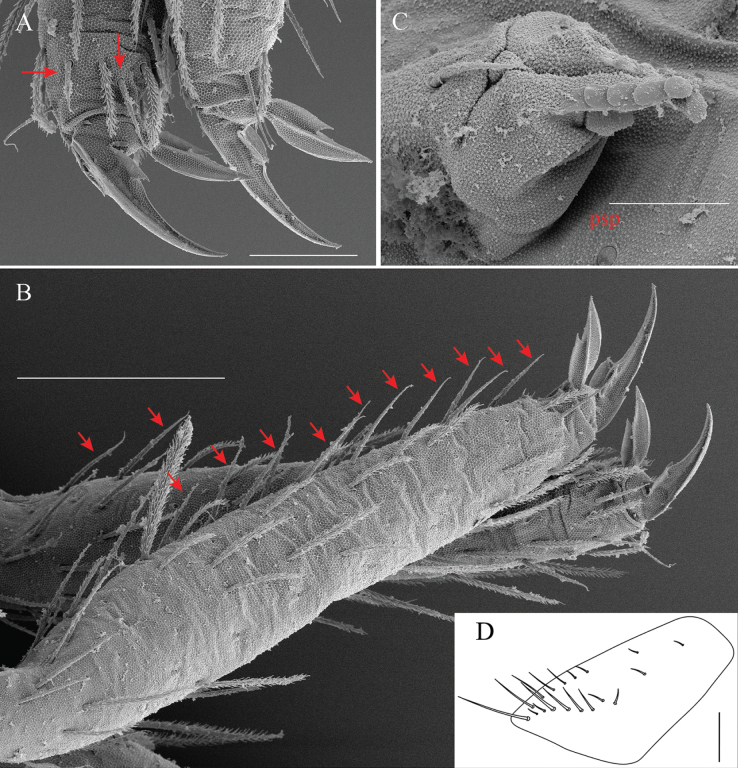
*Alloscopussago* sp. nov. continued, **A.** Claw complex, with red arrow indicates smooth chaetae distally; **B.** Tibiotarsi, showing rows of smooth chaetae and claw morphology; **C.** The tenaculum and psp in close proximity to each other.; **D.** Trochanteral organ. Scale bars: 20 µm (**A, D**); 50 µm (**B**); 10 µm (**C**).

**Figure 7.**
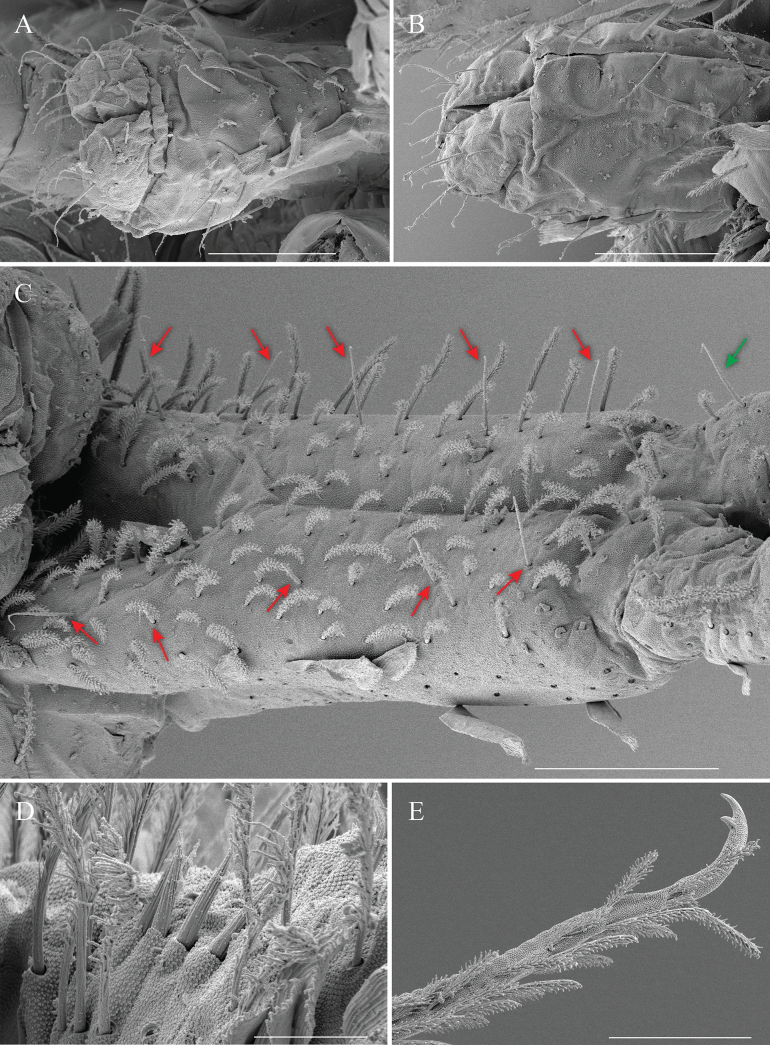
*Alloscopussago* sp. nov. continued, **A.** Ventral tube, posterior view and lateral flap; **B.** Ventral tube, anterior view, and lateral flap; **C.** Dorso-lateral manubrium with red arrows indicating rows of smooth chaetae; green arrow shows a smooth chaeta on dens basally; **D.** Proximal part of dens and spines; **E.** Mucro and distal part of dens. Scale bars: 40 µm (**A, B**); 50 µm (**C**); 10 µm (**D**); 30 µm (**E**).

Figs 1 , 2 , 3 , 4 , 5 , 6 , 7 , Table 1

###### Material examined.

***Holotype***: • female on slides: sample # THA_SJ_ PLG08; Thailand: Phatthalung: Khuankhanun district, 7°44'02.7"N, 99°59'46.2"E, 25 m asl, sago palm forest, by aspirator, coll. S. Jantarit, 25 viii 2020. ***Paratypes***: • collection data same as holotype; on slides: • four samples, • one female and • three subadults, by aspirator (2 specimens) and funnel Berlese extraction (2 specimens).

###### Description.

***Habitus*** (Fig. 2A, B). Medium sized Orchesellidae. Body length (head+trunk) up to 1.5 mm. Scales present on both sides of Ant. I and II, head, body, legs (coxa to femur), ventral tube and furca. Color whitish on all parts of the body without dotted pigments (Fig. 2A). Ocular patch present with small red dark spot (Fig. 2A). Antennal length shorter than the body. Eyes absent. PAO shape irregular (three overlapping ovals) located just below antennal mac (Figs 4E, 5A). Body slender, not bent nor humped at Th. II level.

***Pseudopores*** (Figs 5A, 6C). Present as round flat disks, as large as mac sockets, on various parts of body. Antennae, with psp on tip of Ant. II and III (2 on each segment). Head with 1+1 psp laterally, anterior to PAO; tergites, 1+1 psp near axis, from Th. II to Abd. IV; sternite of Abd. IV with one psp near tenaculum (Figs 5A, 6C). Coxae of legs I and II with 1+1 psp near longitudinal rows of chaetae. Manubrium with 2+2 dorso-apical psp.

***Antennae*** (Fig. 2). Elongated, ~1.8–2.5× cephalic diagonal, 0.35–0.41× body length (head + trunk) (*n* = 4). Ant. I subdivided, Ant. III and IV annulated, except proximal and distal part (Fig. 3A–D). When Ant. II and III fused, segments not annulated. Antennal segment ratio as I(a+b):II:III:IV = 1:1.13:1.60:1.81. Antennal chaetae diverse: ordinary chaetae, S-chaetae (as described in Jantarit and Sangsiri 2020) and scales. Scales oval to rounded, of medium size (7–13 × 14–28 μm), with dense cover of short spicules arranged in more or less regular longitudinal lines; numerous dorsally on Ant. Ib and Ant. II, few on Ant. Ia dorsally; absent on Ant. III and IV (Fig. 3A, B). Sens and sens-like chaetae present on all antennal segments, of 13 morphological types similar to that described by Jantarit and Sangsiri (2020), not re-described in detail here.

Ant. I subdivided into Ia and Ib (Fig. 3A). Ant. Ia shorter than Ib. Ant. Ia dorsally with 2–4 basal mic (type g) and a row of three or four of thick ciliated chaetae (type b); ventrally with four or five pointed smooth mes (type e), a few thick ciliated chaetae (type b) and one hyaline, smooth mic (type m) (Fig. 3A). Ant. Ib dorsally densely covered with scales and ciliated chaetae (types a and b), with a smooth basal mic (type g); apical row with one hyaline, smooth mic (type m), three or four pointed smooth mes (type e) and two or three thin, long, hyaline S-chaetae (type l). Ventrally with a dense cover of ciliated chaetae (type a and b); apical row with three pointed smooth mac (type d) and 8–10 pointed smooth mes (type e or f); lateral side with three or four large swollen, blunt S-chaetae (type h), four or five thin, long hyaline mes (type l) mixed with another three types of S-chaetae (types e, i, k) variously interspersed (Fig. 3A).

Ant. II dorsally with scales mixed with many thick ciliated chaetae (types a and b), distally with 5–7 pointed smooth mes (types e or f); one or two swollen S-chaetae (type h); three thin, long hyaline mes (type l); and six thin hyaline rather short chaetae (type k); basally with three pointed smooth mic (type g). Ventrally with a dense cover of thick ciliated mes (type b) with two pointed smooth mic (type g) basally, mixed with three pointed smooth mac (type d), two large swollen S-chaetae (type h), 10–12 thin, long, hyaline S-chaetae (type l), and 12–15 pointed smooth mes (type e or f) (Fig. 3B).

Ant. III without scale, chaetotaxy with tendency to form whorls of chaetae. Dorsally dense cover with many thick ciliated mes (types b and c) with two pointed smooth mic (type g) basally, mixed with six types of S-chaetae (types e, f, h, i, k, l) variously interspersed. Ventrally with a dense cover of many thick ciliated mes (type b) with two pointed smooth mic (type g) basally, mixed with four types of S-chaetae (types e, f, h, l) variously interspersed (Fig. 3C). Ant. III organ with five sens; sens 1 (type m) longest, hyaline, and acuminate; sens 4 (type i) hyaline, rather long, blunt apically; sens 5 (type j) dark, shortest; sens 2 and 3 (type h) swollen curving rods (Figs 3C, 4A). Antennae sometimes asymmetrical due to the fusion of Ant. III and IV, Ant. III distal chaetae strongly modified when Ant. III and IV fused.

Ant. IV annulated except at tip, without apical bulb, chaetotaxy with tendency to form whorls of chaetae. Dorsally dense cover of many thick ciliated mes (types b and c) mixed with four types of S-chaetae (types e, f, k, l) variously interspersed. Ventrally same as dorsal: dense cover of many thick ciliated mes (types b and c) mixed with four types of S-chaetae (types e, f, k, l) variously interspersed (Fig. 3D). Pin projection on apex absent (Figs 3D, 4B). Subapical organite not distinctly knobbed, apical not enlarged, inserted dorsally near tip of Ant. IV with apical guard chaetae (Figs 3D, 4B).

***Mouthparts*** (Fig. 4C, D). Prelabral and labral chaetae 4/5, 5, 4, all smooth, acuminate, subequal; except chaetae of proximal row slightly longer than others. Four labral papillae, conical, minute (Fig. 4D). Ventral complex of labrum with two slightly asymmetrical multi-toothed combs and a pair of thin, sinuous, unequal tubules below. Maxillary outer lobe with one basal chaeta, simple maxillary palp, four sublobal appendages, all smooth (Fig. 4C). Labial palp with five smooth, acuminate proximal chaetae, five papillae (A = 0, B = 5, C = 0, D = 4, E = 5) and hypostomal chaeta (H) with two accessory appendages (h1 and h2) (Fig. 4C, D). Labial papilla E with lateral process subcylindrical apically, not reaching papilla apex (Fig. 4C). Mandible asymmetrical (right with 4 and left with 5 teeth) on all examined specimens. Molar plate with three or four strong pointed basal teeth. Maxilla with strong tridentate claw, four or five stout ciliated lamellae with two or three well-developed sharp beaks each side opposite to maxilla capitulum, and thin rod, long, bent inwards towards capitulum.

***Ventral head chaetotaxy*** (Fig. 4C). Labial basis as M1m2rel1l2: chaetae M1 ciliated, m2, e and l2 subequal and longest, r and l1 subequal and shortest (Fig. 4C). Postlabial quadrangle (PLQ) with 2+2 weakly serrated chaetae (Fig. 4C). Ventral head densely covered with scales and weakly serrated chaetae.

***Dorsal head chaetotaxy*** (Figs 4E, 5A). Dorsal cephalic chaetotaxy with stable chaetae arrangement (Figs 4E, 5A). ‘An’ series with 8+8 chaetae, all mac; ‘A’ series with 5+5 mac (A0, A2–A5), A1 as mic; ‘M’ series with 3+3 mac (M1–M3), sutural mac with 7+7 mac (S0, S1, S3–6, S6i), and three unnamed mic between series ‘M’ and ‘S’; interocular series with 3+3 chaetae (p as mac, t as mes, r as mic); postsutural area with 3+3 mic (Ps2, 3, 5); postoccipital anterior area with 1+1 mac (Pa5), 1+1 short cephalic tric (Pa6) and 1+1 unnamed mic laterally; postoccipital posterior area with 3+3 mic (Pp3, 5, 6); head laterally with several unnamed mac.

***Tergites*** (Fig. 5A). Dorsal chaetotaxy in Fig. 5A. Formulas for Th. II–Abd. V: psp formula as 1,1/ 1,1,1,1,0; tric formula as 0,0/0,2,3,2,0; ms formula as 1,0/1,0,1,0,0; sens formula as 2,2/1,3,3,3,3; mac formula as 9,7/3,1,3,6,4. Mac arrangement stable; multiplets sensu Szeptycki (1979) present only anterior on Th. II.

Th. II with 4+4 anterior central mac (a5, m2, m4, m4p) and 5+5 posterior mac (p1–3, p5, p2e); with 6+6 mic (m1, m5, 6pi, 1p2, p4, acc.p6), 2+2 mes (p64, p6) and two unnamed mes laterally.

Th. III with 6+6 central mac (p1–3, p1a, a4–5) and 1+1 lateral mac (a6); 6+6 mic (a2, m4, p4–6, m6p) and 4+4 mes (a7, m7, m6 and unnamed mes).

Abd. I with 3+3 central mac (m2–4) and 7+7 mic (a1–3, a5–6, m5, p6) and a row of 2–3+2–3 unnamed mes laterally (only one chaeta shown in Fig. 5A).

Abd. II with 1+1 central mac (m3); 13+13 chaetae (a1–3, a6–7, m3e, m5–6, p4, p6–7 as mic; a5 and m2 as tric) and two unnamed mic laterally, not shown in Fig. 5A.

Abd. III with 1+1 central mac (m3) and 2+2 lateral mac (p6, pm6); 14+14 chaetae (a1–3, a6–8, m3e, m4, p4–6 as mic; a5, m2, m5 as tric); with one unnamed mic and three unnamed mes laterally not shown in Fig. 5A.

Abd. IV with 2+2 central mac (A6, B5); 4+4 lateral mac (E3, E4, Ee, F1); with at least 16+16 chaetae (A2–3, A5, C1, F2, T1, T3 as mic; A6, C4, D3, D4p, T6–7, F3a as mes; T2 and T4 as tric); and at least 12+12 S-like chaetae.

Abd. V with 3+3 central mac (m2–3, p1) and 1+1 lateral mac (m5); with 12+12 chaetae (a1, a3, a5, p1p, p3pi, 3pe, 5pi as mic, a6, ap6, m4–5, m5a as mes); with at least three unnamed mes laterally, not shown in Fig. 5A.

Abd. VI with at least 24+24 ciliated mac mixed with mes, all chaetae not shown in the illustration (Fig. 5A). Dorsal anal valve with two strong and long smooth chaetae, at least 17 chaetae (serrated mes mixed with mac), mic not seen.

***Legs*** (Fig. 6A–D). Covered with ordinary ciliated chaetae (mes–mac), smooth chaetae, and scales; mic not observed.

Subcoxa of leg I with 3+3 mac, subcoxa of leg II with 4–5+4–5 mac and 4+4 mes, subcoxa of leg III with 3–4+3–4 mes anteriorly and 3–4+3–4 mac posteriorly.

Coxa of leg I with two proximal psp, four or five anterior mes and four posterior mac; coxa of leg II with six mac in anterior row, four or five mac in posterior row and one proximal psp in between, at least row of two or three mes posteriorly; coxa of leg III with at least seven chaetae (3 mes anteriorly, 3 mac posteriorly, 8–10 mes in between, psp not seen. Trochanteral organ with 18–21 smooth, straight, unequal spine-like chaetae (Fig. 6D).

Tita of leg III slightly longer than tita of legs I and II. Distal whorl of tita with ten subequal ciliated chaetae, irregularly arranged, and a thin, acuminate, smooth tenent hair. Tita with rows of nine or ten long smooth chaetae internally, two smooth chaetae latero-distally (Fig. 6A, B). Ventro-distal smooth chaeta of tita III thin, erected, pointed, longer than tenent hair or unguiculus. Pretarsal mic minute on anterior and posterior sides.

Ungues outer teeth present; inner edge with paired basal teeth, unpaired teeth absent. Unguiculus ~1/2 as long as inner edge of ungues, slightly swollen baso-internally, pointed apically, with large outer tooth (under light microscope), devoid of inner teeth (Fig. 6A, B).

***Ventral tube*** (Fig. 7A, B). Ventral tube ~1.5× longer than wide; with scales on posterior side (Fig. 7A). Anteriorly with 7+7 subequal ciliated chaetae and one weakly serrated chaeta, (Fig. 7B). Posteriorly with 2+2 smooth chaetae, and 3+3 row of ciliated chaetae mixed with two or three weakly serrated chaetae proximally (Fig. 7A). Lateral flaps with 11+11 smooth chaetae (Fig. 7A, B).

***Furcal complex*** (Figs 6C, 7C–E). Tenaculum with one smooth chaeta and four large teeth of decreasing size from the basal to distal one on each ramus (Fig. 6C).

Ratio of manubrium: dens: mucro = 4.2: 6.44: 1 (*n* = 6).

Mucrodens 1.5–2.0× longer than manubrium (*n* = 6). Manubrium dorsally densely covered with ciliated mes, row of 5+5 smooth chaetae on each side (Fig. 7C). Manubrial plate with 2+2 psp and four ciliated chaetae. Manubrium ventrally densely covered with medium size scales, one short smooth chaeta basally. Dens curved, tapering, constituted of a rather short basal part hardly crenulated, long medial part with well-defined dorsal crenulations and short, thinner, smooth distal part, ~4× mucro length. Dens basally with 1+1 row of four or five finely ciliated spines on inner side (normally with 4+4), sometimes asymmetrical and 1+1 long smooth chaetae latero-externally (Fig. 7C). Laterally dens covered with ciliated mes (Fig. 7C), ventrally with medium-sized elongated scales mixed with ciliated mes; scales absent on distal non-crenulated part (Fig. 7C, E). Mucro bidentate, without basal spine (Fig. 7E).

***Genital plate*** as in *A.whitteni* Jantarit & Sangsiri, 2020. Female with 2+2 smooth mic, one pair on anterior and posterior lobes. Type series without adult males.

###### Remarks.

*Alloscopussago* sp. nov. shows morphological similarity to *A.whitteni* that is found in a cave environment in Phang Nga Province, southern Thailand. Both species share several morphological traits, including the presence of a red dark red eye patch; the absence of an apical pin chaeta on Ant. IV; number of “An” (=8), “A” (=5), “M” (=3) and “S”(=7) series on dorsal head; labial basis formula (M1m2rel1l2); number of processes of papilla E (5); number of spiniform labral papillae (4); number of mac on Th. III (7+7); number of lateral mac on Abd. III (2+2); ungual inner side without unpaired teeth; and presence of smooth chaetae on tibiotarsi. However, *A.sago* sp. nov. differs from the all other blind *Alloscopus* species, including *A.whitteni*, by number of mac on Th. II with only nine mac (vs 10–13 mac) and the presence of 5+5 smooth chaetae on manubrium (vs 3–4+3–4). This new species can also be distinguished from *A.whitteni* by a combination of the following characters: longer antennae (1.8–2.1 vs 1.5); body without pigment (vs orange pigment); 9+9 mac on Th. II (vs 10+10); small number of chaetae on posterior face (7–8 vs 12); and smooth chaetae on manubrium (5+5 vs 4+4). The habitat of the new species is distinctly different, as it is found exclusively within the bark of the sago palm tree, whereas the latter species is confined to cave environments. Diagnostic characters of this new species and all seven closely related blind species are provided in Table 1.

**Table 1.** Morphological comparison among the ten blind *Alloscopus* species.

Characteristics/species	*A.arborealis*	*A.bannaensis*	*A.deharvengi*	*A.jantapasoae* sp. nov.	*A.liuae*	*A.namtip*	*A.sago* sp. nov.	*A.tetracanthus*	*A.thailandensis*	*A.whitteni*
Body length (mm)	0.91–1.51	3	1.4	1.3–1.7	1.6	1.6	1.2–1.5	1.6	1.7	1.7
Ant./head	1.1–1.8	2.2	?	1.59–2.0	1.65	2	1.8–2.5	2.2	?	1.5
Eye patch	absent	present	absent	present dark red	present	present dark red	present dark red	absent to reddish brown	present dark red	Present dark red
Pigmentation	absent	orange pigments	absent	orange dots	absent or light pigment restricted to eye patch	absent	absent	absent	?	orange dots
Ant. IV apical pin chaeta	absent	present	present	absent	present	absent	absent	present or absent	present	absent
PAO shape	irregular	overlapping	irregular	semidivided	semidivided	semidivided	semidivided	semidivided	oval	semidivided
Head chaetotaxy										
An	7	8	5?	9	8(7)	9	8	?	3?	8
A	5 (A0, A2–A5)	5 (A0, A2–A3, A5–A6)	5 (A0, A2–A5)	4 (A0, A2–A4)	5 (A0, A2–A3, A5–A6)	4 (A0, A2–A4)	5 (A0, A2–A5)	4 (A0, A2–A4)	5 (A0, A2–A5)	5 (A0, A2–A5)
M	1 (M1)	3 (M1–M3)	3 (M1–M3)	3 (M1–M3)	3 (M1–M3)	3 (M1–M3)	3 (M1–M3)	3 (M1–M3)	3 (M1–M3)	3 (M1–M3)
S	7 (S0, S1, S3–S6, S6i)	7 (S0, S1, S3–S6, S6i)	7 (S0, S1, S3–S6, S6i)	7 (S0, S1, S3–S6, S6i)	7 (S0, S1, S3–S6, S6i)	7 (S0, S1, S3–S6, S6i)	7 (S0, S1, S3–S6, S6i)	7 (S0, S1, S3–S6, S6i)	7 (S0, S1, S3–S6, S6i)	7 (S0, S1, S3–S6, S6i)
Labial basis	M1m2_e l1_	M1m2el1l2	M1m2?eL1(l1)l2	M1m2_el1l2	?	M1m2rEl1(l2)	M1m2rel1l2	M1(m1)m2rel1l2	M1m2?el1l2	M1m2rel1(l2)
Chaetae of PLQ	weakly serrated	ciliated? Weakly serrated	smooth	weakly serrated	smooth	weakly serrated	weakly serrated	?	smooth	weakly serrated
Lateral processes of papilla E	5	5	4	5	5	5	5	?	3	5
Spiniform labral papillae	4	4	4	4	4	2	4	4	2	4
Chaetotaxy of Th. II	13+13	12+12	12+12	11+11	12+12	11+11	9+9	9–10+9–10	12+12	10+10
central mac	6+6 (a5, m2, m2i, m4, m4i, m4p)	6+6 (a5, m1–2, m4, m4p, m4i)	6+6 (a5, m2, m2i, m4, m4p, m4i)	6+6 (a5, m2, m2i, m4, m4p, m4i)	6+6 (a5, m1–2, m4, m4p, m4i)	6+6 (a5, m2, m2i, m4, m4p, m4i)	4+4 (a5, m2, m4, m4p)	4–5+4–5 (a5, m2, m4, (m4p), m4i)	6+6 (a5, m2, m2i, m4, m4p, m4i)	5+5 (a5, m2, m4, m4p, m4i)
posterior mac	7+7 (p1–p5, 1p, 2a)	6+6 (p1–p3, p5, 1p, 1p2, p2e)	6+6 (p1–2, p4–6, 1p)	5+5 (p1–3, p2e, p5)	6+6 (p1–p3, p5, 1p, 1p2, p2e)	5+5 (p1–3, p2e, p5)	5+5 (p1–3, p2e, p5)	4–5+4–5 (p1–2, (p3), p4–5)	6+6 (p1–2, p4–6, 1p)	5+5 (p1–3, p2e, p5)
Chaetotaxy of Th. III	8+8	8+8	6+6?	7+7	7+7	7+7	7+7	6+6?	7+7	7+7
central mac	7+7 (p1–3, p1a, a4–5, and unnamed mac)	6+6 (p1–3, p1a, a4–5)	6+6 (p1–3, p1a, a4–5)	6+6 (p1–3, p1a, a4–5)	6+6 (p1–3, p1a, a4–5)	6+6 (p1–3, p1a, a4–5)	6+6 (p1–3, p1a, a4–5)	6+6 (p1–3, p1a, a4–5)	6+6 (p1–3, p1a, a4–5)	6+6 (p1–3, p1a, a4–5)
lateral mac	1+1 (a6)	2+2 (a6, m6)	?	1+1 (a6)	1+1 (a6)	1+1 (a6)	1+1 (a6)	?	1+1 (a6)	1+1 (a6)
Lateral mac on Abd. III	1(pm6)	2(pm6, p6)	2(pm6, p6)	2(pm6, p6)	1(pm6)	2(pm6, p6)	2(pm6, p6)	2(pm6, p6)	2(pm6, p6)	2(pm6, p6)
Chaetotaxy of Abd. IV	6+6	7+7	?	7+7	5+5	6+6	6+6	?	?	6+6
central mac	2+2 (A6, B5)	2+2 (A6. B5)	2+2 (?)	3+3 (A6, B6, C2)	2+2 (A6, B5)	2+2 (A6, B5)	2+2 (A6, B5)	2+2 (A6, B5)	2+2 (?)	2+2 (A6, B5)
lateral mac	4+4 (E1, E3, F3, Ee10)	5+5 (E3–E4, F1–F3)	?	4+4 (E1, E3–4, Ee)	3+3 (E3, F1, F3)	4+4 (E1, E3–4, Ee)	4+4 (E1, E3–4, Ee)	?	?	4+4 (E1, E3–4, Ee)
Tergal ms (from Th. II–Abd. V)	1,0,0,0,1,0,0	1,0,1,0,1,0,0	?	1,0,1,0,1,0,0	1,0,1,0,1,0,0	1,0,1,0,1,0,0	1,0,1,0,1,0,0	?	?	1,0,1,0,1,0,0
sens (from Th. II–Abd. V, elongated one not included)	2,2,1,3,3,4,3	2,2,1,3,3,3,3	?	2,2,1,3,3,3,3	2,2,1,3,3,3,3	2,2,1,3,3,3,3	2,2,1,3,3,3,3	?	?	2,2,1,3,3,3,3
Ungual inner unpaired teeth	0	0	0	0–1 (tiny)	1	1–2	0	0	1–2	0
Smooth chaetae on tibiotarsi	absent	absent	present	absent	present	absent	present	present	absent	present
Smooth chaetae on trochanteral organ	19–22	19–32 (spine-like chaetae)	17	16–22	13–18 (spine-like chaetae)	25–32	18–21	15	15	12–20
Chaetae on ventral tube										
anterior face	6–7+6–7 (4c, 2–3se)	8–11	?	6+6	6–9	9+9	7+7	8+8	?	7–9+7–9
posterior face	4–5 + 4–5 (2c, 2–3se)	many	?	10–11+10–11	11–17	23	7–8+7–8	16+16?	?	12+12?
lateral flap	7+7	13–16	?	11+11	9–15	12+12	11+11	?	?	11–12+11–12
Smooth chaetae on manubrium	4+4	3+3	4+4	3+3	4+4	4+4	5+5	3+3	4+4	4+4
Smooth chaetae on lateral anal valves	0	0	2	0	1	0	2	2–3	0	3
Number of spines on dens	5–8	5–6	3–5	3–5	3–5	4–6	4–5	4–7	3–6	4–6
Ecology	canopy and forest floor	litter or on leaves of forest floor	humus and mosses	cave	litter	cave	inside the bark of sago palm trees	leaf litter, forests, tea field	leaf litter, soil, roots, tree bark	cave
Distribution	Philippines	China	Papua New Guinea	Thailand	China	Thailand	Thailand	Australasia, South Asia, Pacific	Thailand	Thailand
Source	Alviola et al. 2024	Zhang et al. 2020	Mari-Mutt 1985; Cipola et al. 2016	This study	Zhang et al. 2020	Jantarit and Sangsiri 2020	This study	Börner 1906; Mari-Mutt 1977, 1982; Prabhoo 1971; Yoshii and Suhardjono 1989; Cipola et al. 2016	Mari-Mutt 1985; Cipola et al. 2016	Jantarit and Sangsiri 2020

? indicates no information. Note: Chaetotaxy of Th. II for *A.tetracanthus* follows Mari-Mutt (1977: 244, 1982: 90) and Prabhoo (1971: 34).

###### Etymology.

The species name is derived from the habitat in which this new species was found, the sago palm (*Metroxylonsagu* Rottb.), a true sago palm typically found in lowland freshwater swamps. The name emphasizes the presence of springtails in the sago palm forests, which are native to southern Thailand, and underscores the ecological significance of the sago palm that serves not only as a major source of starch for commercial purposes but also as a habitat for numerous species, including the newly described Collembola species.

###### Ecology.

The new species is exclusively found on the old bark of the sago palm (*Metroxylonsagu* Rottb) at the lower trunk level (Figs 1B, 2F). The sago palm population in this area is dense and is located within a protected zone managed by the local community at Ban Huay Pru, Khaun Khanun Subdistrict, Khaun Khanun District, Phatthalung Province, designated for the cultivation and harvesting of sago palm. The sago palm patch spans approximately 4.75 hectares (47,500 square meters) and is surrounded by agricultural areas, including rice fields, orchard plantations, and residential communities.

##### ﻿ Alloscopus jantapasoae

Taxon classificationAnimalia

Jantarit, Nilsai & Manee sp. nov.

03BF1C65-DAAA-58D8-A599-26FA780957C2

https://zoobank.org/D413A604-BF51-47CA-9B9E-4F0A63CBBF25

**Figure 8.**
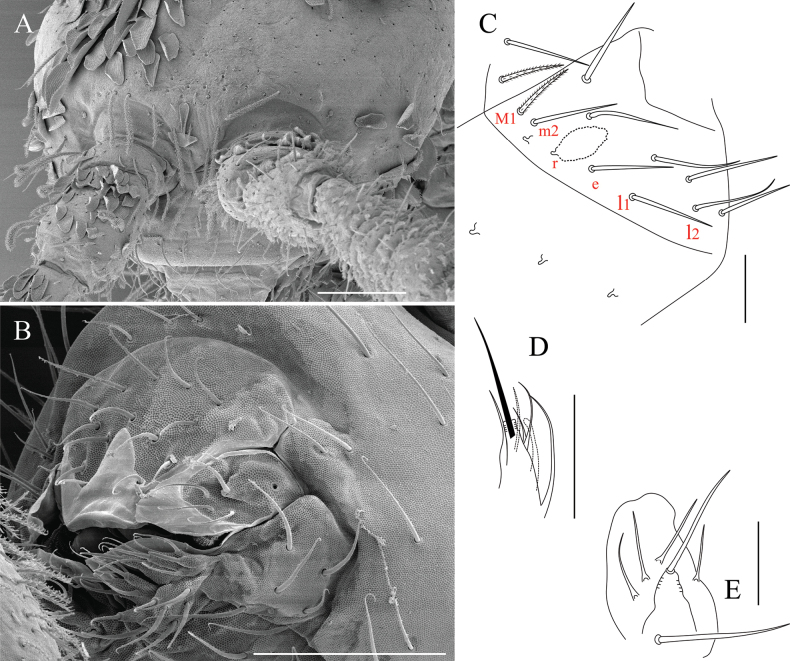
*Alloscopusjantapasoae* sp. nov. **A.** Anterior part of head; **B.** Mouthparts show prelabral and labral chaeta, labial palp, and maxillary outer lobe; **C.** Labial basis; **D.** Papillae E of labial palp (right side); **E.** Maxillary outer lobe. Scale bars: 50 µm (**A, B**); 20 µm (**C, D, E**).

Figs 1 , 2 , 5 , 7 , 8 ; Table 1

###### Material examined.

***Holotype***: • female on slides: sample # THA_SJ_TRG03; Thailand: Trang: Huai Yot District, Pak Chaem subdistrict; Tham Khao Tang Lon (note: “tham” = “cave” in Thai): 7°42'19.1"N, 99°41'12.2"E, 170 m asl, by aspirator, coll. S. Jantarit, 21 xii 2019. ***Paratypes***: • collection data same as holotype; on slides: • seven samples, • one female and • six subadults, by aspirator.

###### Description.

***Habitus*** (Fig. 2C, D). Medium sized Orchesellidae. Body length (head + trunk) up to 1.7 mm. Scales present on both sides of Ant. I–II, on both sides of head, body, legs (coxa to femur), ventral tube and furca. Color whitish in ethanol with orange dotted pigments on antennae, head, body, legs and furca (Fig. 2C). Dark red ocular patch presence. Antennal length shorter than the body. Eyes absent. PAO shape irregular (three overlapping ovals) located just below antennal mac. Body slender, not bent nor humped at level of Th. II.

**Figure 9.**
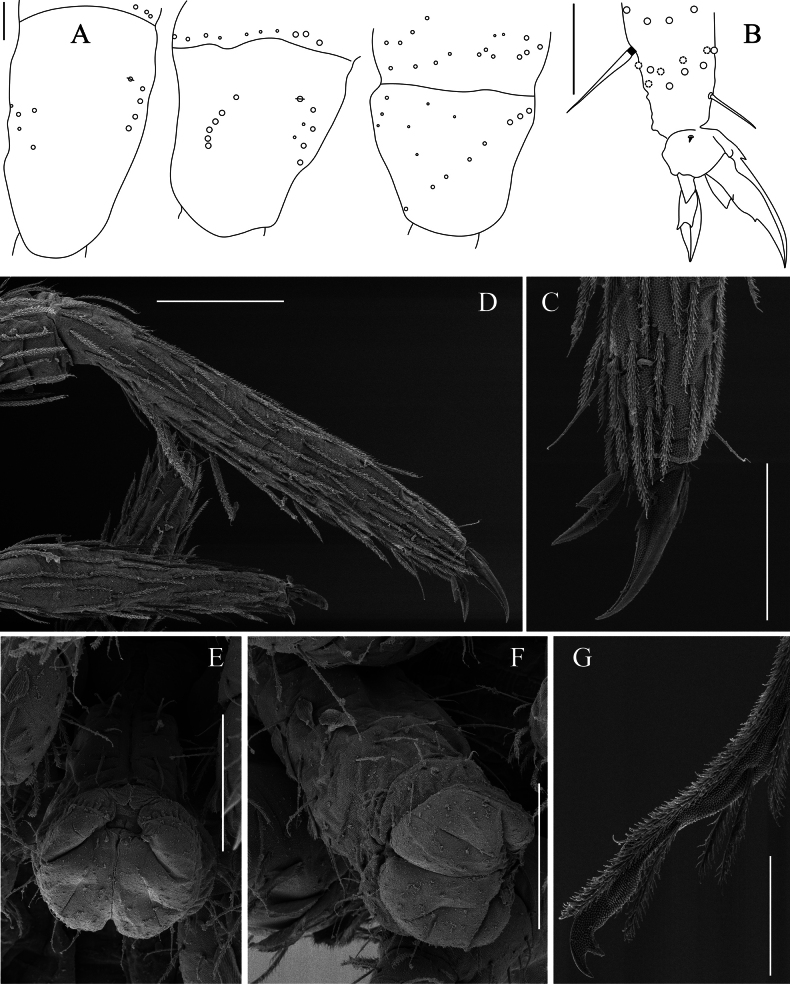
*Alloscopusjantapasoae* sp. nov. (continued). **A.** Outer chaetotaxy of coxae I–III (left side); **B, C.** Distal part of tibiotarsi and claw complex; **D.** Tita and claw morphology; **E.** Ventral tube, anterior view, and lateral flap; **F.** Ventral tube, posterior view, and lateral flap; **G.** Mucro and distal part of dens. Scale bars: 20 µm (**A, B, G**); 50 µm (**D, E, F**); 30 µm (**C**).

***Pseudopores*** (Figs 5B, 9A) present as round flat disks, as large as mac sockets, present on various parts of body: antennae, head, tergites, coxae and manubrium. Antennae with psp located on tip of Ant. II and III (2 on each segment). Head with 1+1 psp laterally, anterior to PAO. Tergites, 1+1 psp near axis, from Th. II to Abd. IV (Fig. 5B). Sternite of Abd. IV with one psp near tenaculum. Coxae of legs I–II with 1+1 psp near longitudinal rows of chaetae (Fig. 9A). On manubrium with 2+2 dorso-apical ones.

***Antennae*** (Fig. 8A). Slightly longer, ~1.59–2.0× cephalic diagonal, and 0.28–0.41× body length (head + trunk) (*n* = 6). Ant. I subdivided, Ant. III and IV annulated, except proximal and distal part (Fig. 2). When Ant. II and III fused, segments not annulated. Antennal segment ratio as I(a+b):II:III:IV = 1:1.1: 1.25: 1.6. Antennal chaetae diverse: ordinary chaetae, S-chaetae (as described in Jantarit and Sangsiri 2020) and scales. Scales oval to rounded, of medium size (7–13 × 14–28 μm), with dense cover of short spicules arranged in more or less regular longitudinal lines; numerous dorsally on Ant. Ib and Ant. II, few on Ant. Ia dorsally; absent on Ant. III and IV. Sense and sens-like chaetae present on all antennal segments, of 13 morphological types similar to that described by Jantarit and Sangsiri (2020), not re-described in detail here.

Ant. I subdivided into Ia and Ib (Fig. 8A). Ant. Ia shorter than Ib. Ant. Ia dorsally with three basal mic (type g) and few thick ciliated chaetae (type b); ventrally with three basal mic (type g), three pointed smooth mes (type e), one hyaline, smooth mic (type m) and three thick ciliated chaetae (type b). Ant. Ib dorsally densely covered with scales and ciliated chaetae (types a and b), with a smooth basal mic (type g); apical row with two or three hyaline, smooth mic (type m), one pointed smooth mes (type e) and one thin, long, hyaline S-chaetae (type l). Ventrally with a dense cover of ciliated chaetae (types a and b); apical row with three pointed smooth mac (type d) and three or four pointed smooth mes (type e or f); lateral side with two or three large swollen, blunt S-chaetae (type h), four or five thin, long hyaline mes (type l) mixed with other three types of S-chaetae (types e, i, k) variously interspersed.

Ant. II dorsally with scales mixed with many thick ciliated chaetae (types a and b), distally with 5–7 pointed smooth mes (type e or f); one or two swollen S-chaetae (type h); four thin, long hyaline mes (type l); one or two thin hyaline rather short chaetae (type k); basally with three pointed smooth mic (type g). Ventrally with a dense cover of thick ciliated mes (type b) with two pointed smooth mic (type g) basally, mixed with two pointed smooth mac (type d), one short, pointed, rather bent apically (type 7), three or four large swollen S-chaetae (type h), 8–10 thin, long hyaline S-chaetae (type l), and 8–10 pointed smooth mes (type e or f).

Ant. III without scale, chaetotaxy without tendency to form whorls of chaetae. Dorsally dense cover with many thick ciliated mes (types b and c) with two pointed smooth mic (type g) basally, mixed with six types of S-chaetae (types e, f, h, i, k, l) variously interspersed. Ventrally with a dense cover of many thick ciliated mes (type b) with two pointed smooth mic (type g) basally, mixed with four types of S-chaetae (types e, f, h, l) variously interspersed. Ant. III organ with five sens; sens 1 (type m) longest, hyaline, and acuminate; sens 4 (type i) hyaline, rather long, blunt apically; sens 5 (type j) dark, shortest; sens 2 and 3 (type h) swollen curving rods. Antennae sometimes asymmetrical due to the fusion of Ant. III and IV, Ant. III distal chaetae strongly modified when Ant. III and IV fused.

Ant. IV annulated, except at tip, without apical bulb, chaetotaxy with tendency to form whorls of chaetae. Dorsally dense cover with many thick ciliated mes (types b and c) mixed with four types of S-chaetae (types e, f, k, l) variously interspersed. Ventrally dense cover with many thick ciliated mes (types b and c) mixed with four types of S-chaetae (types e, f, k, l) variously interspersed. Pin projection on apex not seen. Subapical organite not distinctly knobbed, apical not enlarged, inserted dorsally near the tip of Ant. IV with apical guard chaetae.

***Mouthparts*** (Fig. 8B, D, E). Prelabral and labral chaetae 4/5, 5, 4, all smooth, acuminate, subequal; except chaetae of proximal row slightly longer than others (Fig. 8B). Four labral papillae, conical, minute. Ventral complex of labrum with two slightly asymmetrical multi-toothed combs and a pair of thin, sinuous, unequal tubules below. Maxillary outer lobe with one basal chaeta, simple maxillary palp, four sublobal appendages, all smooth (Fig. 8B, E). Labial palp with five smooth, acuminate proximal chaetae and five papillae (A=0, B=5, C=0, D=4, E=5), and hypostomal chaeta (H) with two accessory appendages (h1 and h2). Labial papilla E with lateral process subcylindrical apically, reaching papilla apex (Fig. 8B, D). Mandible asymmetrical (right with 4 and left with 5 teeth) on all examined specimens. Molar plate with three to four strong pointed basal teeth. Maxilla with strong tridentate claw, four or five stout ciliated lamellae with two or three well-developed sharp beaks each side opposite to maxilla capitulum, and thin rod, long, bent inwards towards capitulum.

***Ventral head chaetotaxy*** (Fig. 8C). Labial basis as M_1_m_2__el_1_l_2:_ chaetae M1 ciliated, m_2_, l_1_ and l_1_ subequal and longest, r absent, maybe as scale (Fig. 8C). Postlabial quadrangle (PLQ) with 2+2 weakly serrated chaetae. Ventral head with a dense cover of scales and weakly serrated chaetae.

***Dorsal head chaetotaxy*** (Figs 5B, 8A). Dorsal cephalic chaetotaxy with stable chaetae arrangement (Fig. 5B). ‘An’ series with 9+9 chaetae, all mac, ‘A’ series with 4+4 mac (A0, A2–A4), A1 as mic; ‘M’ series with 3+3 mac (M1–M3), sutural mac with 7+7 mac (S0, S1, S3–6, S6i), and three unnamed mic between series ‘M’ and ‘S’; interocular series with 3+3 chaetae (p as mac, t as mes, r as mic); postsutural area with 3+3 mic (Ps2–3 and 5); postoccipital anterior area with 1+1 mac (Pa5), 1+1 short cephalic tric (Pa6) and 1+1 unnamed mic laterally; postoccipital posterior area with 3+3 mic (Pp3 and Pp5–6); head laterally with several unnamed mac (Figs 5B, 8A).

***Tergites*** (Fig. 5B). Dorsal chaetotaxy illustrated in Fig. 5B. Formulas for Th. II–Abd. V: psp formula as 1,1/ 1,1,1,1,0; tric formula as 0,0/0,2,3,2,0; ms formula as 1,0/1,0,1,0,0; sens formula as 2,2/1,3,3,3,3; mac formula as 11,7/3,1,3,7,4. Mac arrangement stable; multiplets sensu Szeptycki (1979) present only anterior on Th. II.

Th. II with 6+6 anterior central mac (a5, m2, m2i, m4, m4i, m4p) and 5+5 posterior mac (p1–3, p5, p2e); with 3+3 mic (1p2, p4, acc.p6), 2+2 mes (p64, p6) and two unnamed mes laterally.

Th. III with 6+6 central mac (p1–3, p1a, a4–5) and 1+1 lateral mac (a6); 9+9 mic (a2, m1, m4, p4–6, p2a, m6p, acc.p6) and 4+4 mes (a7, m6–7, and unnamed mes).

Abd. I with 3+3 central mac (m2–4) and 7+7 mic (a1–3, a5–6, m5, p6) and a row of 2–3+2–3 unnamed mes laterally, not shown in the illustration (Fig. 5B).

Abd. II with 1+1 central mac (m3); 12+12 chaetae (a1–3, a6–7, m3e, m5–6, p4, p6 as mic; a5 and m2 as tric).

Abd. III with 1+1 central mac (m3) and 2+2 lateral mac (p6, pm6); 13+13 chaetae (a1–3, a6–8, m3e, m4, p4–5 as mic; a5, m2, m5 as tric) with two unnamed mes laterally, not shown in the illustration (Fig. 5B).

Abd. IV with 3+3 central mac (A6, B6, C2); 4+4 lateral mac (E3, E4, Ee, F1); with at least 18+18 chaetae (A2–3, A5, B3–4, C1, D1–2, E1, F2, F3a, T1, T3 as mic; C4, D3, D4p, T6–7 as mes; T2 and T4 as tric); and at least 9+9 S-like chaetae (most chaetae lost during slide preparation).

Abd. V with 3+3 central mac (m2–3, p1) and 1+1 lateral mac (m5); with 13+13 chaetae (a1, a3, a5, p1p, p3, p3pi, 3pi, 5pi as mic, a6, ap6, p4–5, m5a as mes) with at least two or three unnamed mes laterally, not shown in Fig. 5B.

Abd. VI with 3+3 ciliated mic and at least 30+30 ciliated mac mixed with mes, all chaetae not shown in Fig. 5B.

Dorsal anal valve without smooth chaetae, several serrated mes mixed with mac, mic not seen.

***Legs*** (Fig. 9A–D). With ordinary ciliated chaetae of various lengths (mes to mac), smooth chaetae and scales; mic not seen. Tita of leg III slightly longer than tita of legs I and II.

Subcoxa of leg I with 3+3 mac, subcoxa of leg II with 3+3 mac and a row of 6–7+6–7 mes, subcoxa of leg III with 3–4+3–4 mac and 12–13+12–13 mes (Fig. 9A).

Coxa of leg I with one proximal psp, four or five anterior mes and four posterior mac; coxa of leg II with six mac in anterior row, four mac and two mes in posterior row and one proximal psp in between; coxa of leg III with 11 or 12 mes anteriorly and three mac posteriorly, psp not seen (Fig. 9A). Trochanteral organ with 17–22 smooth, straight, unequal spine-like chaetae.

Tita distal whorl with ten subequal ciliated chaetae, irregularly arranged, and a thin, acuminate, smooth tenent hair. Tita without rows of long smooth chaetae internally (Fig. 9D). Ventro-distal smooth chaeta of tita III thin, erected, pointed, longer than tenent hair or unguiculus (Fig. 9B–D). Pretarsal mic minute on anterior and posterior sides.

Ungues outer tooth present; inner edge with paired basal teeth, leg III with a tiny unpaired tooth (sometimes absence), legs I and II without unpaired inner teeth. Unguiculus ~1/2 as long as inner edge of ungues, slightly swollen baso-internally, pointed apically, with large outer tooth (under light microscope), devoid of inner teeth (Fig. 9B–D).

***Ventral tube*** (Fig. 9E, F). Ventral tube ~1.5–1.7× longer than wide; with scales on posterior side (Fig. 9F). Anteriorly with 6+6 ciliated chaetae, with approximately the same size (Fig. 9E). Posteriorly with 1+1 smooth chaetae and 9–10+9–10 weakly ciliated chaetae (Fig. 9F). Lateral flaps with 11+11 thin, smooth chaetae of unequal size (Fig. 9F).

***Furcal complex*** (Fig. 9G). Tenaculum with one smooth chaeta and four large teeth of decreasing size from the basal to distal one on each ramus.

Ratio of manubrium: dens: mucro = 4.7: 7.7: 1 (*n* = 8).

Mucrodens 1.58–2.2× longer than manubrium (*n* = 8). Manubrium dorsally densely covered with ciliated mes, with a row of 3+3 smooth chaetae on each side. Manubrial plate with 2+2 psp and four ciliated chaetae. Manubrium ventrally densely covered with medium size scales, chaetae not seen, except basally with 1+1 short smooth chaetae. Dens curved, tapering, constituted of a rather short basal part hardly crenulated, long medial part with well-defined dorsal crenulations and short, thinner, smooth distal part, smooth section ~4× as long as mucro. Dens basally with 1+1 row of 3–5+3–5 finely ciliated spines on inner side (normally with 4+4), sometimes asymmetrical and 1+1 long smooth chaetae latero-externally. Laterally dens covered with ciliated mes, ventrally with medium size elongated scales mixed with ciliated mes; scales absent on distal non-crenulated part. Mucro bidentate, without basal spine (Fig. 9G).

***Genital plate*** as in *A.whitteni* (Jantarit and Sangsiri 2020: fig. 6G). Female with 2+2 smooth mic, one pair on anterior and posterior lobes.

###### Remarks.

*Alloscopusjantapasoae* sp. nov. differs from the all other blind *Alloscopus* species by having 3+3 central mac on Abd. IV (vs 2+2 in all known blind species). The new species is most similar to *A.namtip* Jantarit & Sangsiri, 2020 from Thailand in the number of mac on head of ‘An’ series; number and homology of mac on Th. II (11+11); and due to both species were collected from cave habitats. However, it differs from *A.namtip* in having shorter antennal length (1.2–1.6 vs 2); in the presence of orange pigments (vs absence); number of spiniform labral papillae (4 vs 2); number of mac on Abd. IV (7 vs 6); number of unequal inner unpaired teeth (0–1(tiny) vs 1–2); number of smooth chaetae on trochanteral organ (17–23 vs 25–32); number of chaetae on anterior face of ventral tube (6 vs 9), on posterior face of ventral tube (10–11 vs 23) and number of chaetae on lateral flap of ventral tube (11 vs 12). Diagnostic characters of this new species and all seven closely related blind species are provided in Table 1.

###### Etymology.

*Alloscopusjantapasoae* is named in honor of Ms. Kanchana Jantapaso, a member of our research team who has made significant contributions to the study of cave fauna in Thailand and assisted in the collection and analysis of cave fauna both across the country and in the laboratory.

###### Ecology.

The new species was collected and only known from the dark zone of a cave on ground floor with small patch of bat guano. Seven caves in the surrounding area, including Tham Khao Chang Hai—which is known for having one of the richest cave faunas in the country—were surveyed for Collembola. However, none of the specimens of this species were found in these caves, suggesting a high degree of endemism to Tham Khao Tang Lon.

### ﻿Identification key and distribution of the world species of *Alloscopus*

**Table:** 

1	Mucro with a basal spine	**2**
–	Mucro without a basal spine	**3**
2	Eyes 2+2; Abd. I with 2+2 mac	***A.spinosus* (Prabhoo, 1971) (India)**
–	Eyes 3+3; Abd. I with 3+3 mac	***A.strebeli* Winter, 1966 (Peru and Ecuador)**
3	Eyes absent	**4**
–	Eyes present	**13**
4	Tibiotarsi I–II with smooth inner chaetae	**5**
–	Tibiotarsi I–II without smooth inner chaetae	**9**
5	Th. II with 12+12 mac	**6**
–	Th. II with 9–11+9–11 mac	**7**
6	Eye patch absent; unguis without unpaired teeth	***A.deharvengi* (Mari-Mutt, 1985) (Papua New Guinea)**
–	Eye patch present, unguis with unpaired teeth	***A.liuae* Zhang, 2020 in Zhang et al. 2020 (China)**
7	‘A’ series on dorsal head with 4 chaetae (A5 absent); manubrium with 3+3 smooth chaetae	***A.tetracanthus* (Börner, 1906) (Australasia, South Asia, SE Asia, Papua New Guinea, and Pacific)**
–	‘A’ series on dorsal head with 5 chaetae (A0, A2–A5); manubrium with 4–5+4–5 smooth chaetae	**8**
8	Body without pigmentation; Th. II with 9+9 mac; manubrium with 5+5 smooth chaetae	***A.sago* sp. nov. (Thailand)**
–	Body with orange dots; Th. II with 10+10 mac; manubrium with 4+4 smooth chaetae	***A.whitteni* Jantarit & Sangsiri, 2020 (Thailand)**
9	Ant. IV apical pin chaeta present	**10**
–	Ant. IV apical pin chaeta absent	**11**
10	Labrum with 2 spiniform papillae; Th. III with 8+8 mac; unguis with 1 or 2 unpaired teeth; manubrium with 4+4 smooth chaetae	***A.thailandensis* (Mari-Mutt, 1985) (Thailand)**
–	Labrum with 4 spiniform papillae; Th. III with 7+7 mac; unguis without unpaired teeth; manubrium with 3+3 smooth chaetae	***A.bannaensis* Zhang, 2020 in Zhang et al. 2020 (China)**
11	Body with oranges dots; Abd. IV with 7+7 mac	***A.jantapasoae* sp. nov. (Thailand)**
–	Body without pigment; Abd. IV with 6+6 mac	**12**
12	‘A’ series on dorsal head with 4 chaetae (A5 absence); Th. II with 11+11 mac; Th. III with 7+7	***A.namtip* Jantarit & Sangsiri, 2020 (Thailand)**
–	‘A’ series on dorsal head with 5 chaetae (A0, A2–A5);Th. II with 13+13 mac; Th. III with 8+8	***A.arborealis* Alviola, Lucañas & Jantarit, 2024 (Philippines)**
13	Eyes 2+2; unguiculus without an outer tooth	***A.aspinosus* (Prabhoo, 1971) (India)**
–	Eyes 1+1; unguiculus with an outer tooth	**14**
14	Head mac Pa5 present	**15**
–	Head mac Pa5 absent	**16**
15	Head mac A5 present and M3 absent; Abd. II with 1+1 lateral mac (m5); trochanteral organ with up to 25 spines. Dens dorsally with a row of 5–14 spines	***A.tenuicornis* (Börner, 1906) (Indonesia, Papua New Guinea, Philippines, Micronesia, and Hawaii)**
–	Head mac A5 absent and M3 present. Abd II without lateral mac; trochanteral organ with 30–40 spines. Dens dorsally with two rows of 27–55 spines	***A.multispinatus* (Mari-Mutt, 1982) (Indonesia and Papua New Guinea)**
16	Head ‘M’ series with 1+1 mac (M1); Th. II–III with 13+13 and 7+7 mac, respectively; manubrium dorsally with 3+3 smooth chaetae	***A.fallax* Yoshii & Suhardjono, 1992 (Indonesia)**
–	Head ‘M’ series with 2+2 mac (M1–2). Th. II–III with 11+11 and 6+6 mac, respectively; manubrium dorsally with 4+4 or 5+5 smooth chaetae	***A.yosiius* (Mari-Mutt, 1985) (Indonesia)**

## Supplementary Material

XML Treatment for Alloscopus sago

XML Treatment for Alloscopus jantapasoae

## ﻿Additional information

### Use of AI

No use of AI was reported.

### Author ORCIDs

Sopark Jantarit https://orcid.org/0000-0001-8401-2946

Nongnapat Manee https://orcid.org/0009-0007-4016-9176

Areeruk Nilsai https://orcid.org/0000-0002-4052-6328

Natrada Mitpuangchon https://orcid.org/0009-0005-4677-5578

Awatsaya Pimsai https://orcid.org/0000-0003-3974-7059

### Data availability

All of the data that support the findings of this study are available in the main text.

## References

[B1] Absolon K Ksenemann M (1942) Troglopedetini. Vergleichende Studie über eine altertümliche höhlenbewohnende Kollembolengruppe aus den dinarischen Karstgebieten. Studien aus dem Gebiete der Allgemeinen Karstforschung, der Wissenschaftlichen Höhlenkunde, der Eiszeitforschung und den Nachbargebieten 16: 5–57.

[B2] Alviola MS Lucañas CC Lit Jr IL Soto-Adames FN Jantarit S (2024) A new canopy-dwelling species of the genus *Alloscopus* Börner (Collembola: Orchesellidae: Heteromurinae) from Mt. Makiling, Philippines. Zootaxa 5045(2): 281–295. 10.11646/zootaxa.5405.2.8 38480384

[B3] Börner C (1906) Das System der Collembolen nebst Beschreibung neuer Collembolen des Hamburger Naturhistorischen Museums. Mitteilungen aus den Naturhistorischen Museum in Hamburg, 23: 147–188.

[B4] Cipola NG Oliveira FG Morais JW Bellini BC (2016) The Heteromurinae Absolon & Ksenemann (Collembola, Entomobryidae): A review of the genera status and diagnoses, keys for species of *Alloscopus* Börner and Heteromurtrella Mari-Mutt and description of a new species. Zootaxa 4084(2): 151–186. 10.11646/zootaxa.4084.2.1 27394258

[B5] Fjellberg A (1999) The labial palp in Collembola. Zoologischer Anzeiger 237: 309–330.

[B6] Gisin H (1967) Espèces nouvelles et lignées évolutives de *Pseudosinella endogées* (Collembola). Memorias e Estudos do Museu Zoologico da Universidade de Coimbra 301: 1–21.

[B7] Godeiro NN Ding Y Cipola NG Jantarit S Bellini BC Zhang F (2023) Phylogenomics and systematics of Entomobryoidea (Collembola): Marker design, phylogeny and classification. Cladistics 39(2): 1–15. 10.1111/cla.12521 36583450

[B8] Jantarit S Sangsiri T (2020) Two new species of *Alloscopus* from caves in Thailand, with a key to world species of the genus (Hexapoda: Collembola). The Raffles Bulletin of Zoology 35: 48–60.

[B9] Jantarit S Bedos A Deharveng L (2016) An annotated checklist of the Collambolan fauna of Thailand. Zootaxa 4169(2): 301–360. 10.11646/zootaxa.4169.2.4 27701300

[B10] Mari-Mutt JA (1977) The taxonomic status of *Alloscopus* and redescription of its two species. The Pan-Pacific Entomologist 53(4): 241–249.

[B11] Mari-Mutt JA (1982) A new species of Heteromurus (Alloscopus) from Papua New Guinea and descriptive notes for the other species of the subgenus (Collembola: Entomobryidae: Orchesellinae). Pacific Insects 24(1): 84–94.

[B12] Mari-Mutt JA (1985) Three new species of Heteromurus (Alloscopus) and descriptive notes for species of the subgenus (Collembola: Entomobryidae). The Florida Entomologist 68(2): 335–346. 10.2307/3494370

[B13] Prabhoo NR (1971) Soil and litter collembola of South India. Oriental Insects 5(1): 1–46. 10.1080/00305316.1971.10433988

[B14] Ren Y Li Z Zhang F (2018) A new species of *Dicranocentrus* Schött from Hainan (China) with a key to the Chinese species of the genus (Collembola, Entomobryidae). ZooKeys 762: 59–68. 10.3897/zookeys.762.23926 PMC5990583 29887738

[B15] Soto-Adames FN (2008) Postembryonic development of the dorsal chaetotaxy in *Seiradowlingi* (Collembola, Entomobryidae); with an analysis of the diagnostic and phylogenetic significance of primary chaetotaxy in Seira. Zootaxa 1683(1): 1–31. 10.11646/zootaxa.1683.1.1

[B16] Szeptycki A (1979) Morpho-systematic studies of Collembola. IV. Chaetotaxy of the Entomobryidae and its phylogenetical significance. Polska Akademia Nauk, Zakład Zoologii Systematycznej i Doświadczalnej, Państwowe Wydawnictwo Naukowe, Warszawa, Kraków, 218 pp.

[B17] Winter C (1966) Beiträge zur Kenntnis der neotropischen Collembolenfauna. Entomological Zeitung 76: 165–169.

[B18] Yoshii R Suhardjono YR (1992) Notes on the Collembolan fauna of Indonesia and its Vicinities. II. Collembola of Irian Jaya and Maluku Islands. Acta Zoologica Asiae Orientalis 2: 1–52.

[B19] Zhang F Deharveng L (2015) Systematic revision of Entomobryidae (Collembola) by integrating molecular and new morphological evidence. Zoologica Scripta 44(3): 298–311. 10.1111/zsc.12100

[B20] Zhang F Bellini BC Soto-Adames FN (2019) New insights into the systematics of Entomobryoidea (Collembola: Entomobryomorpha): first instar chaetotaxy, homolog and classification. Zoological Systematics 44(4): 249–278. 10.11865/zs.201926

[B21] Zhang F Cipola NG Pan ZX Ding Y (2020) New insight into the systematics of Heteromurinae (Collembola: Entomobryidae: Heteromurinae) with special reference to *Alloscopus* and *Sinodicranocentrus* gen.n. Arthropod Systematics & Phylogeny 78(1): 1–16. 10.26049/ASP78-1-2020-01

